# Betulinic acid induces apoptosis and suppresses metastasis in hepatocellular carcinoma cell lines in vitro and in vivo

**DOI:** 10.1111/jcmm.13964

**Published:** 2018-11-12

**Authors:** Wei Wang, Yang Wang, Mingxing Liu, Yifan Zhang, Tao Yang, Dongsheng Li, Yinpeng Huang, Qing Li, Guang Bai, Linlin Shi

**Affiliations:** ^1^ Department of General surgery First Affiliated Hospital of Jinzhou Medical University Jinzhou China; ^2^ Graduate School Jinzhou Medical University Jinzhou China; ^3^ Department of Internal Medicine Third Affiliated Hospital of Jinzhou Medical University Jinzhou China; ^4^ Library Shandong University Weihai China

**Keywords:** apoptosis, betulinic acid, hepatocellular carcinoma, metastasis

## Abstract

Hepatocellular carcinoma (HCC) is a high incidence and mortality malignant tumour globally. Betulinic acid (BA) is a pentacyclic triterpenoid with potential pro‐apoptotic activities which widely found in many plants. In this study, we determined the effects of BA on proliferation, apoptosis, invasion, and metastasis in HCC cell lines and on tumour growth and pulmonary metastasis in mice. The results suggested that BA could inhibit cell viability and proliferation of HCC cell lines including HepG2, LM3, and MHCC97H. In addition, BA induced apoptosis of HepG2 cells characterised condensed nuclei and nuclear fragmentation. Moreover, western blot analysis showed that BA‐induced apoptosis associated with increasing of pro‐apoptotic protein Bax and cleaved caspase‐3 and decreasing of anti‐apoptotic protein Bcl‐2. Meanwhile, BA also reduced the reactive oxygen species (ROS) level. Furthermore, BA also significantly inhibited HCC growth in vivo and blocked pulmonary metastasis of HCC by regulating the metastasis‐related proteins including MMP‐2, MMP‐9, and TIMP2 without obvious toxicity. In all, the present study suggested that BA might be a promising anti‐HCC drug candidate by inhibiting proliferation, inducing apoptosis, and blocking metastasis.

## 1 INTRODUCTION

Liver cancer is a worldwide health problem, and hepatocellular carcinoma (HCC) is the most common primary liver malignancy.^1^ As reported in the United States cancer statistics data in 2017, about 40 710 new liver cancers and 28 920 deaths were estimated to occur. Higher incidence and death in males than females were observed for 2.5:1 and 2.1:1 respectively.^2^ In the United States, the HCC incidence showed a declining trend, however, it is still rising in many developing regions such as China, eastern and southern Asia, etc.^3^, ^4^ The incidence of HCC varies greatly by geographic region and associated with various risk factors including hepatitis B and hepatitis C viruses infection, cirrhosis (chronic liver damage induced by inflammation and fibrosis), alcohol abuse, older age, male sex, metabolic syndrome, and aflatoxin B exposure.^5^, ^6^ Multiple treatment approaches have opted in different stages of HCC for survival benefits. For example, in the early‐stage, resection, liver transplantation, or local ablation were available and shows survival benefits; however, in the developed stages only chemoembolisation (intermediate HCC) and chemotherapy (advanced HCC) show benefits.^7^ However, the current therapies are commonly associated with recurrence, metastasis, side effects, and drug resistance.^8^ Hence, the safer and efficacious novel agents are urgently needed for the treatment of HCC.

Furthermore, HCC is highly malignant with metastatic potential, and its poor prognosis and mortality at advanced stages mostly contributed to the recurrence and metastasis.^9^, ^10^ Pulmonary metastasis is the most common type of metastasis at advanced HCC, which cause poor prognoses and HCC‐related death.^11^ Metastasis is a complex and dynamic process associating a serial of biological and pathological events, including cancer cell motility, intravasation into circulation, transit in the blood or lymph, and extravasation to the new site, termed the invasion‐metastasis cascade.^12^, ^13^ Emerging evidence has demonstrated that epithelial‐mesenchymal transition (EMT) plays a vital role in invasion and metastasis of malignant tumour, and indicated that the EMT processes promoted and mediated by tumour‐associated matrix metalloproteinases (MMPs) such as MMP2 and MMP9.^14^ The activities of MMPs were regulated by the tissue inhibitors of metalloproteinases (TIMPs). It has been considered that the overexpression of TIMPs induced down‐regulation of MMPs and suppressed the invasive and metastatic abilities of tumour cells.^15^, ^16^

Nowadays, natural original products are one of the main sources of anticancer drug research for their varied biological activity. BA is a pentacyclic triterpenoid which widely exists in various plants, fruits, and vegetables, especially rich in the birch tree bark (*Betula sp*.) (up to 2.5%).^17^, ^18^ In the last decades, the biological activities and medicinal properties of BA have been intensively investigated; the results revealed that BA exhibits a plethora of beneficial properties, including anti‐tumour, anti‐angiogenic, anti‐inflammatory, anti‐fibrotic, anti‐HIV, and hepatoprotective effect.^19^ Furthermore, the most concerned activity of BA is anti‐tumoral activity currently, it has reported that BA shows significant cytotoxic on various tumour cell lines originating from lung, colon, prostate, and ovary, and in animal models in vivo.^19^, ^20^ However, only a few papers on liver cancer were published. Eichenmuller et al have determined that BA‐induced apoptosis of hepatoblastoma cells, but its resistant to HCC cells line HepG2.^21^ However, Yang et al further suggested that BA inhibits proliferation and induces apoptosis of HepG2 cell, and inhibits tumour growth in HCC mice.^22^ In the current study, we evaluated the effect of BA on proliferation, apoptosis, invasion, migration, and metastasis in HCC cell lines and tumour growth in vivo. Moreover, the inhibitory effect on pulmonary metastasis and toxicity of BA were also investigated in vivo.

## 2 MATERIALS AND METHODS

### 2.1 Reagents and antibodies

Betulinic acid (BA) was obtained from Sigma‐Aldrich (St Louis, MO) (purity > 98%). MTT, Hoechst 33258, Rhodamine 123 and DCFH‐DA were obtained from Beyotime Biotech (Shanghai, China). Annexin V‐FITC apoptosis detection kit was purchased from eBioscience (San Diego, CA). The primary antibodies against Bcl‐2, Bax, Cleaved Caspase‐3, MMP‐2, MMP‐9, TIMP‐2, and β‐actin were purchased from Cell Signaling Technology (MA, USA). Mouse monoclonal antiKi‐67 was obtained from Merck‐Millipore (MA, USA).

### 2.2 Cell lines and animals

The HCC cell lines HepG2, LM3, and MHCC97H were obtained from ATCC (MD, USA). These cells were propagated in DMEM added with 10% FBS and 1% antibiotics (penicillin 100 U/mL and streptomycin 100 mg/mL) at 5% CO_2_ at 37°C. NOD/SCID mice (20 ± 2 g, 6 ~ 8 weeks) were purchased from Beijing Huafukang bioscience CO. INC. (Beijing, China). The mice were housed in a standard laboratory condition (temperature 22 ± 2°C and humidity 50% ~ 60%). All animal experiments were performed in accordance with the guidelines of the Experimental Research Institute of Jinzhou Medical University.

### 2.3 Cell viability assay

MTT method was used to test the cell viability of BA on HepG2, LM3, MHCC97H cells. Briefly, the exponentially growing cell suspensions were planted in 96‐well culture plates at 3 × 10^3^ cells/well. Overnight, the supernatants were removed, and the cells treated with a series increasing concentrations of BA (2.5‐40 μm) and vehicle (0.1% DMSO) for 24, 48, and 72 hours, respectively. After that, 20 μL MTT (5 mg/mL) was added and incubated at 37°C for additional 2~4 hours. Then, the supernatants were removed, the formazan dissolved in 150 μL DMSO for 10 minutes. The absorbance was recorded at 490 nm by a microplate reader (Bio‐Rad). The inhibition rates (%) were calculated.

### 2.4 Colony formation assay

The exponentially growing cell suspensions of HepG2 and LM3 were planted in 6‐well plates (100‐300 cells/well). Overnight, the cells were treated with BA (5, 10, 20 μm) and vehicle (0.1% DMSO) and cultured for 2 weeks. The supernatants were removed and washed with PBS twice. Then the colonies were fixed with methanol for 15 minutes and stained with crystal violet solution (0.5%) for 30 minutes. Finally, the colonies with >10 cells were recorded under a microscope.

### 2.5 Morphological analysis

Hoechst 33 258 staining was conducted to identify whether the BA inducing morphological change such as cell body shrinkage, chromatin condensation, and emerging apoptotic bodies associated with apoptosis in HepG2 cells.^23^ Briefly, HepG2 cells were planted in 6‐well plates at 1 × 10^5^ cells/well and incubation for 24 hours. The cells treated with BA (5, 10, 20 μm) and vehicle (0.1% DMSO) for 48 hours. Finally, the cells were washed with cold PBS and stained with the Hoechst 33258 solutions (5 μg/mL) following the manufacturer's instructions. Then nuclear morphology of cells was observed under a fluorescence microscopy (Zeiss, Germany).

### 2.6 Apoptosis analysis

The numbers of apoptotic cells induced by BA were determined by flow cytometry. Briefly, HepG2 cells were planted in 6‐well plates at 1 × 10^5^ cells/well. Then the cells incubated with BA (5, 10, 20 μm) and vehicle (0.1% DMSO) for 24 hours, the cells were collected and washed with PBS. Then the Annexin V‐FITC apoptosis detection kit was used to determine the apoptosis rates following to manufacturer's instructions by flow cytometry.

### 2.7 Migration and invasion analysis

Transwell chamber was applied to investigate the migration and invasion ability of BA on HepG2 cells. Briefly, HepG2 cells were planted in 24‐well plates at 1 × 10^4^ wells in the upper chamber with 100 μL serum‐free medium, and 600 μL of medium supplemented 10% FBS was added in the lower chamber. Different concentrations of BA (5, 10, 20 μm) and vehicle (0.1% DMSO) were added in the upper and lower chambers, respectively. After migration for 48 hours, the un‐migrated cells in the upper chamber were removed, and the migrated cells on the lower surface were fixed with ice‐cold methanol for 10 min and stained with crystal violet (0.5%) for 30 minutes protect from light. The migrated cells in five randomly selected fields were quantitated under a light microscopy. For the invasion assay, the medium diluted Matrigel (60 μL/well) were added into the upper transwell chamber to form membrane on the upper surface, 600 μL of medium supplemented 10% FBS was added in the lower chamber. The cells were seeded at 1 × 10^4^ wells in the upper chamber with 100 μL serum‐free medium, and treatment with different concentrations of BA (0, 5, 10, 20 μ mol L^−1^) for 24 h. Then, the cells in the upper chamber were removed, and the cells on the underside were fixed with ice‐cold methanol for 10 minutes and then stained with crystal violet (0.5%). The migrated cells were quantitated and photographed under a light microscope.

### 2.8 Adhesion assay

MTT assay was used to determine the effect of BA on adhesion ability of HepG2 cells. Briefly, the Matrigel was coated on 96‐well plates (25 μL/well) at 37°C for 24 hours, and then the wells were blocked with 3% bovine serum albumin. The HepG2 cells treatment with BA (5, 10, 20 μm) and vehicle (0.1% DMSO), and then detached from culture by trypsin. The treated cells added into the wells (5 × 10^4^ cells/well) and incubated for 24 hours. After that, the unattached cells were removed, 180 μL DMEM, and 20 μL MTT (5 mg/mL) were added to each well, and then incubated for additional 2 ~ 4 hours. Then the formazan dissolved with 150 μL DMSO for 10 minutes. The absorbance was recorded at 490 nm by a microplate reader (Bio‐Rad). The adhesive ratios (%) were calculated.

### 2.9 Mitochondrial membrane potential (∆Ψm) assay

The mitochondrial transmembrane potential changing of HepG2 cells affected by BA were detected using rhodamine 123. Briefly, the HepG2 cells were planted in 6‐well plates at 1 × 10^5^ cells/well. After incubation overnight, the cells treated with BA (5, 10, 20 μm) and vehicle (0.1% DMSO) for 36 hours. Then the cells were washed with cold PBS and added Rh123 (5 μg/mL) incubated for 30 minutes protect from light and then determined by FCM.

### 2.10 Reactive oxygen species measurement

The intracellular ROS level of HepG2 cells affected by BA was quantified by DCFH‐DA method.^24^ Briefly, the HepG2 cells were planted in 6‐well plates at 1 × 10^6^ cells/well. After incubation overnight, the cells treated with BA (5, 10, 20 μm) and vehicle (0.1% DMSO) for 48 hours. Then the cells added 10 μmol/L of DCFH‐DA incubated for 30 minutes in dark at 37°C. Then the cells were washed with PBS and harvested. The fluorescence was measured by flow cytometry.

### 2.11 Western blot assay

The western blot assay was conducted as described previously.^25^ In brief, the HepG2 cells were planted in 6‐well plates at 1 × 10^5^ cells/well. After incubation overnight, the cells treated with BA (5, 10, 20 μm) and vehicle (0.1% DMSO) for 24 hours. Then cells were washed with PBS and lysed in RIPA buffer. The protein concentration was determined by BCA method. Non‐specific binding was blocked with 5% skimmed milk for 1.5 hours, and the membranes were incubated with diluted primary antibodies (Bcl‐2, Bax, Cleaved caspase 3, TIMP2, MMP‐2, MMP‐9, and β‐actin) overnight at 4°C and in the secondary antibody for 1 hour at room temperature. The signals were determined by Amersham prime ECL Plus detection system (Pittsburgh, PA).

### 2.12 Experimental HCC and pulmonary metastasis model

Eighteen NOD/SCID mice were implanted subcutaneously with 100 μL HepG2 cells suspensions (1 × 10^7^ cells/mouse) into the left dorsal. When the visible tumours at the injection sites grown up to ~50 mm^3^, the mice were randomly divided into three groups (n = 6), and intraperitoneally injection (i.p.) with BA 5, 10 mg/kg (dissolved in corn oil) or equal corn oil (vehicle) dosed at 0.1 ml/10 g of body weight for 18 d, respectively. The tumour volume was measured every 3 days as follow formula: Tumour volume (mm^3^) = 0.5 × L × W^2^, the L is the length and W is the width. Finally, all the mice were killed, and the tumours were excised and photographed and weighted using to evaluate the antitumour effect of BA, the blood and organs (heart, liver, lung, spleen, and kidney) were collected using to evaluate the potential toxicity of BA.

For pulmonary metastasis assay, the NOD/SCID mice were injected with 100 μL HepG2 cells suspensions (1 × 10^6^ cells/mouse) via tail vein. Seven days later, the mice were randomly divided into three groups (n = 3), and intraperitoneally injection (i.p.) with BA 5, 10 mg/kg (dissolved in corn oil) or equal corn oil (vehicle) dosed at 0.1 ml/10 g of body weight for 18 d, respectively. Finally, the mice were killed and the lungs were separated and the nods were photographed and counted.

### 2.13 Immunohistochemistry

Immunohistochemistry (IHC) staining of tumour sections was performed with a commercial detection kit according to the standard instruction. Briefly, the tumours sections (4 μm) were deparaffinised and rehydrated through xylene and graded alcohols and then immersed in target retrieval solution in water bath for 30 minutes. The endogenous peroxidase was blocked with 3% H_2_O_2_ for 15 minutes, and the non‐specific bindings were blocked with goat serum for 50 minutes. Then, the slides stained with primary antibodies (Ki‐67 and MMP‐2) and secondary antibody polymer HRP successively. The slides then stained with DAB and counterstained with methyl green. Images were taken with a Leica microscope.

### 2.14 Toxicity evaluation

The blood and organs (heart, liver, lung, spleen, and kidney) were collected at the end of the HCC experiment. The blood used for routine analysis and biochemical analysis. The organs tissues were stained with H&E for histopathologic assay.

### 2.15 Statistical analysis

All data were presented as means ± SD. Statistical analysis was conducted by one‐way ANOVA with spss 13.0. Statistical significance was accepted at *P* values *<*0.05.

## 3 RESULTS

### 3.1 BA inhibits HCC cell lines viability

**Figure 1:**
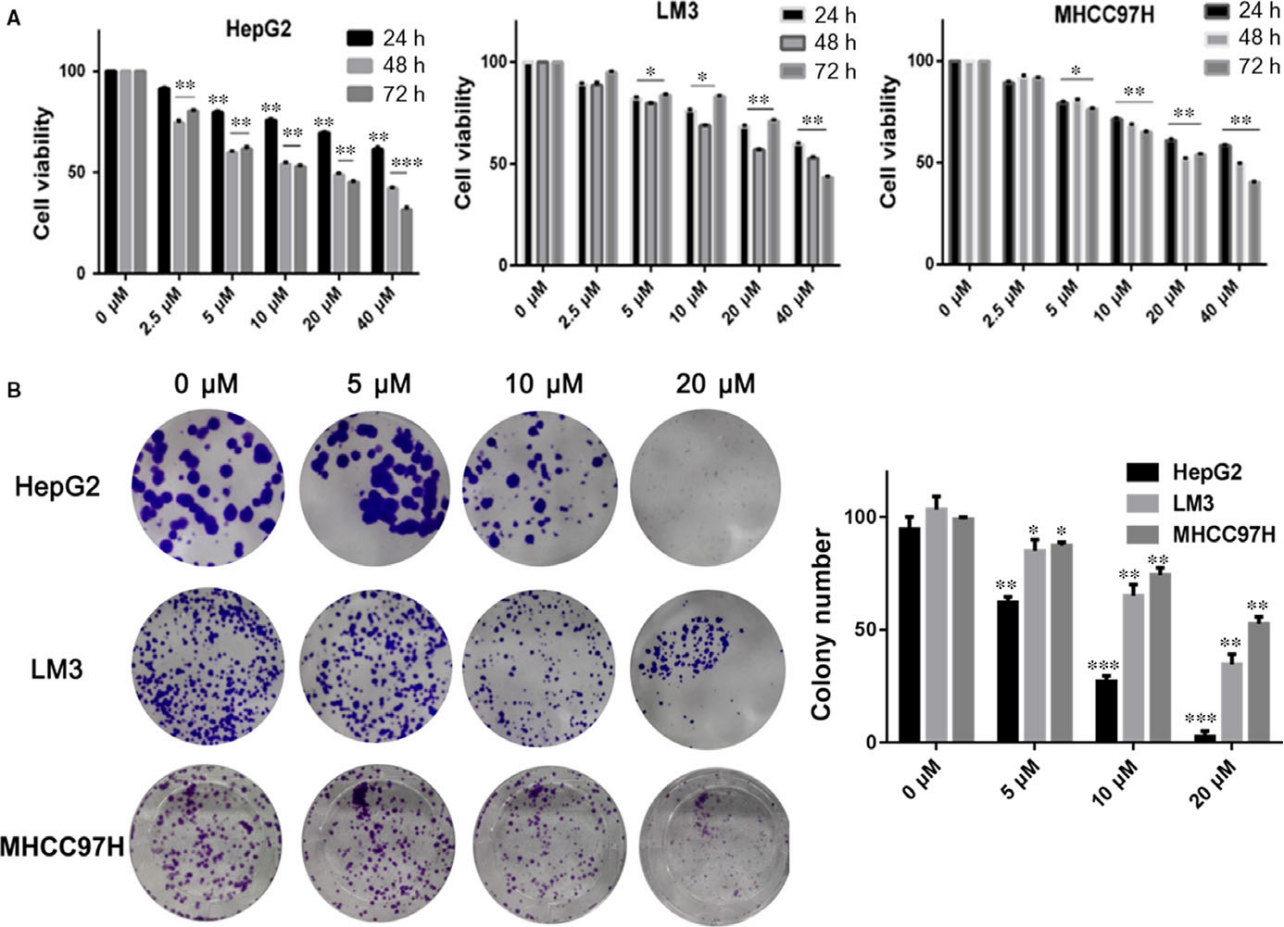
The inhibitory effects of BA on HCC cell lines viability. A, The cell viability of HepG2, LM3, and MHCC97H cells treated with BA (2.5‐40 μm) and vehicle (0.1% DMSO) for 24, 48, and 72 h, respectively. B, The colony formation inhibition of BA (5, 10, 20 μm) and vehicle (0.1% DMSO) in HepG2, LM3, and MHCC97H cells for 14 d. The surviving colonies with >10 cells were counted. *P < 0.05, **P < 0.01, ***P < 0.001 vs Control (0 μm) group

In order to determine whether BA has direct inhibition effects on HCC cell lines, the cell viability and clonogenic assays were conducted on HCC cell lines. The cell viability caused by BA was tested by MTT method. As shown in Figure 1A, the viability of HepG2, LM3, and MHCC97H cells decreased after treated with BA for 24, 48, and 72 hours, respectively. These data indicated that BA inhibited the viability of HCC cell lines in a time‐ and concentration‐dependent manner. Furthermore, we also measured the inhibitory effect of BA on proliferation by clonogenic assay. As shown in Figure 1B, BA significantly inhibited the clone formation of HepG2, LM3, and MHCC97H cells in a concentration‐dependent manner. Moreover, the HepG2 cells were more sensitive to BA than LM3 and MHCC97H cells. In all, both results indicated that BA showed a strong inhibition effect of viability and proliferation on various HCC cell lines.

### 3.2 Betulinic acid induces HepG2 cells apoptosis

**Figure 2:**
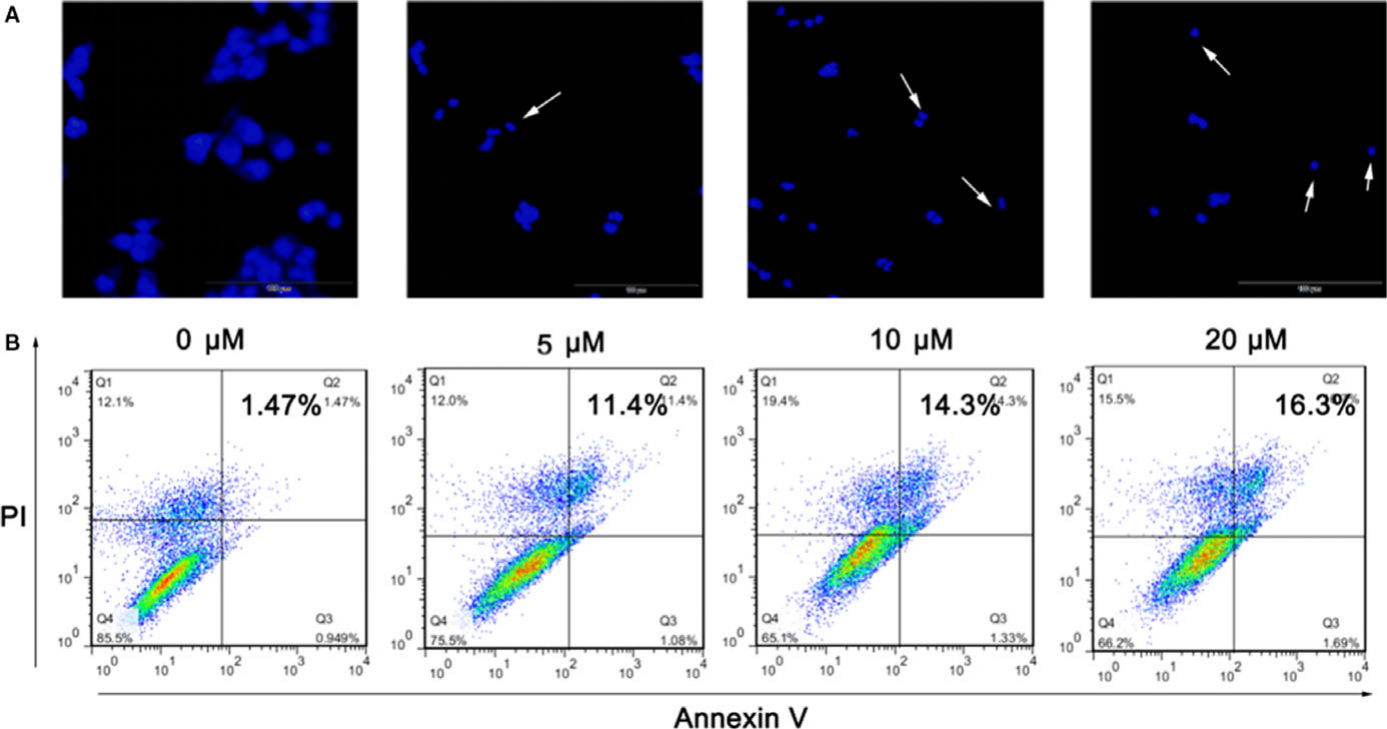
BA induces HepG2 cells apoptosis. A, The fluorescence microscopic appearance of HepG2 cells stained by Hoechst 33258 after treatment with BA (5, 10, 20 μm) and vehicle (0.1% DMSO) for 48 h. B, The apoptosis rate of HepG2 cells determined by flow cytometry after treatment with BA (5, 10, 20 μm) and vehicle (0.1% DMSO) for 24 h. *P < 0.05, **P < 0.01, ***P < 0.001 vs Control (0 μm) group

In order to explore whether BA induced HepG2 apoptosis, Hoechst staining, and flow cytometry assay was conducted. As shown in Figure 2A, BA treatment induced HepG2 cells apoptosis characterised condensed nuclei and nuclear fragmentation. The apoptosis rate which determined by flow cytometry showed that BA treatment increased the apoptosis rate significantly (Figure 2B).

### 3.3 BA induces apoptosis of HepG2 cells via the mitochondrial apoptotic pathway

**Figure 3:**
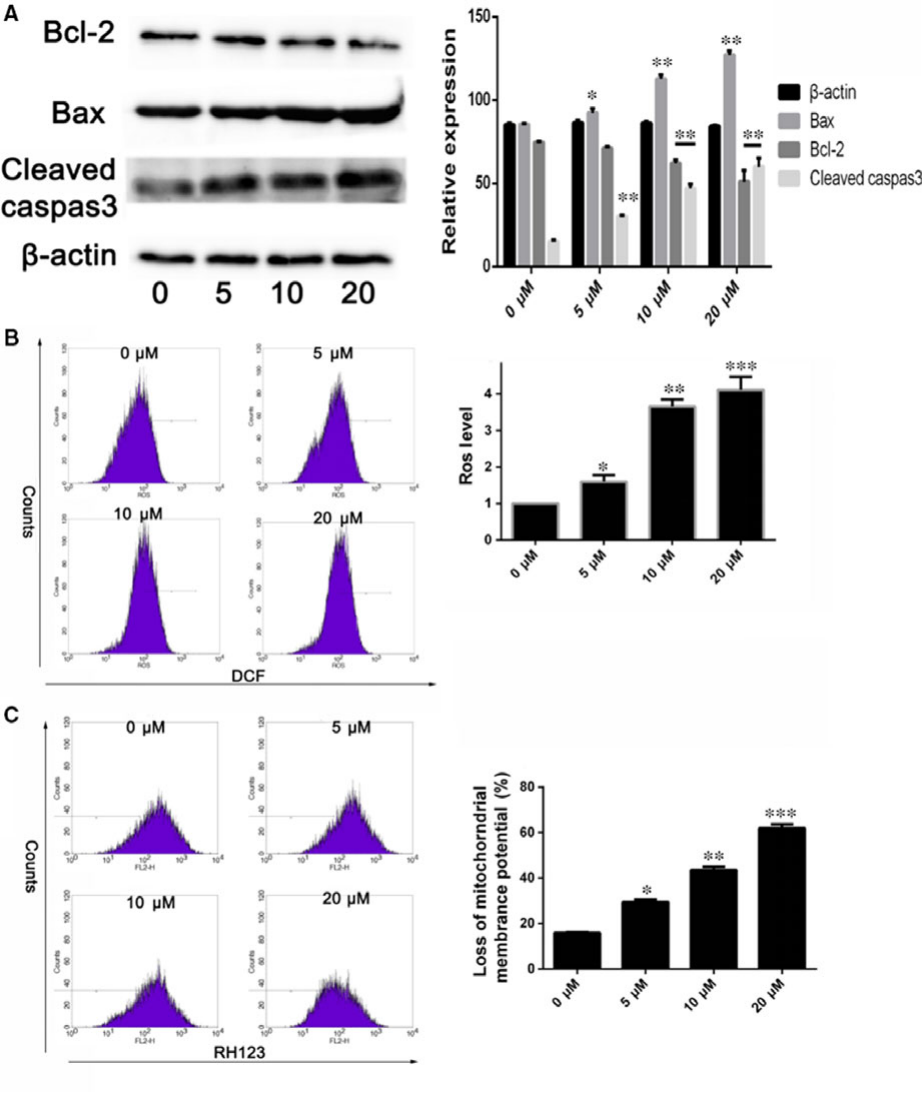
Betulinic acid induces apoptosis of HepG2 cells. A, The levels of anti‐apoptotic Bcl‐2 and pro‐apoptotic Bax and cleaved caspase‐3 in HepG2 cells detected by western blot after treatment with BA (5, 10, 20 μm) and vehicle (0.1% DMSO) for 24 h. B, The change of mitochondrial membrane potential in HepG2 cells induced by BA (5, 10, 20 μm) and vehicle (0.1% DMSO) for 36 h determined by flow cytometry. C, The intracellular ROS level changing in HepG2 cells after treatment with BA (5, 10, 20 μm) and vehicle (0.1% DMSO) were determined by flow cytometry. *P < 0.05, **P < 0.01, ***P < 0.001 vs Control (0 μm) group

In order to clarify the mechanism of apoptosis induced by BA on HepG2 cells, the apoptosis‐related proteins level, the mitochondrial transmembrane potential (∆Ψm) and intracellular ROS level were investigated. The levels of anti‐apoptotic Bcl‐2 and pro‐apoptotic Bax and cleaved caspase‐3 in HepG2 cells detected to further explore the characterisation of BA on apoptosis by western blot. As shown in Figure 3A, BA significantly decreased the level of anti‐apoptotic Bcl‐2 in a concentration‐dependent manner, whereas significantly increased the levels of Bax and cleaved caspase‐3 (*P < *0.01). The damage of mitochondria and loss of ∆Ψm play an important role in the intrinsic apoptotic pathway.^26^ So we further tested the disruption of mitochondrial membrane potential induced by BA. As shown in Figure 3B, 29%, 43%, and 62% of cells lost mitochondrial membrane potential after treatment with BA at 5, 10, and 20 μ mol L^−1^ for 36 hours, respectively. Moreover, ROS was considered to be able to trigger apoptosis in the mitochondria.^27^ So the intracellular ROS levels were detected by DCFH‐DA method after BA treatment. As shown in Figure 3C, BA treatment induced stronger fluorescence intensity in HepG2 cells significantly compared to control cells, which suggested an enhanced ROS level. Taken together, these results indicated that BA induces the apoptosis of HepG2 might be via the mitochondrial apoptotic pathway.

### 3.4 BA inhibits HepG2 cells migration, invasion, and adhesion

**Figure 4:**
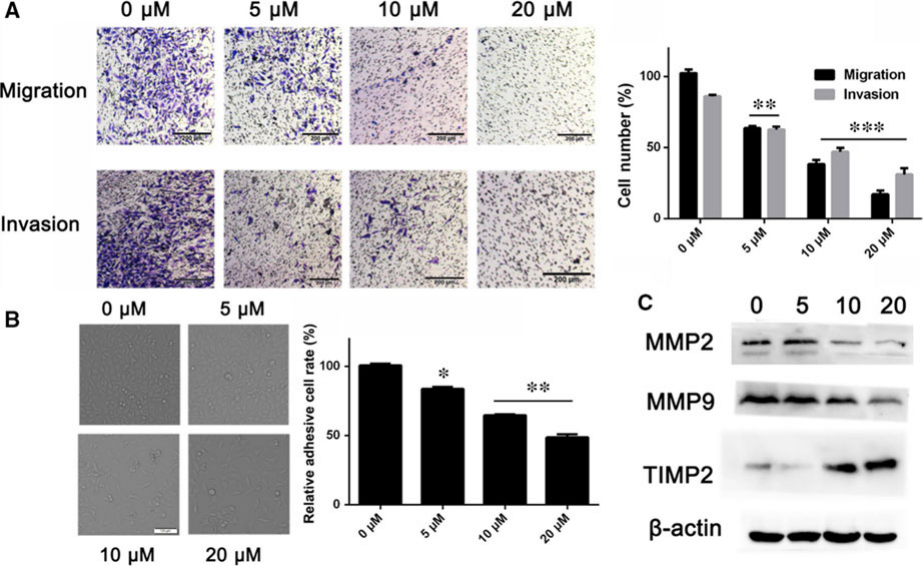
Betulinic acid inhibits HepG2 cells migration, invasion, and adhesion. A, The effect of BA on migration and invasion ability of HepG2 cells conducted by transwell chamber. B, The effect of BA on adhesion ability of HepG2 cells determined by MTT method. C, The effect of BA on metastasis‐related proteins MMP‐2, MMP‐9, and TIMP2 determined by western blot. *P < 0.05, **P < 0.01, ***P < 0.001 vs Control (0 μm) group

The migration and invasion abilities of tumour cells were demonstrated to be one of the pivotal steps in various tumour metastasis.^28^ Therefore, we further investigated the effect of BA on invasion and migration abilities on HepG2 cell. The results as shown in Figure 4A, BA significantly inhibits migration and invasion of HepG2 cell in a concentration‐dependent manner (*P < *0.01). Similar results were observed in the adhesion assay, BA significantly decreased the adhesive ratios (%) of HepG2 cells in a dose‐dependent manner (Figure 4B). Moreover, the published evidence has demonstrated that the MMPs such as MMP‐2 and MMP‐9 play an important role on metastasis of malignant tumours for degrading the basement membrane and promoting invasion.^29^ Therefore, we investigated the expression of MMP‐2 and MMP‐9, and TIMP2 in HepG2 cell, which is involved in BA‐mediated migration and invasion. As shown in Figure 4C, the expression of MMP‐2 and MMP‐9 were decreased and its inhibitor TIMP2 was increased after treatment with B. Altogether, the above results suggested that BA showed a strong inhibitory effect on HepG2 cells migration and invasion.

### 3.5 BA reduces HepG2 tumour growth in vivo

**Figure 5:**
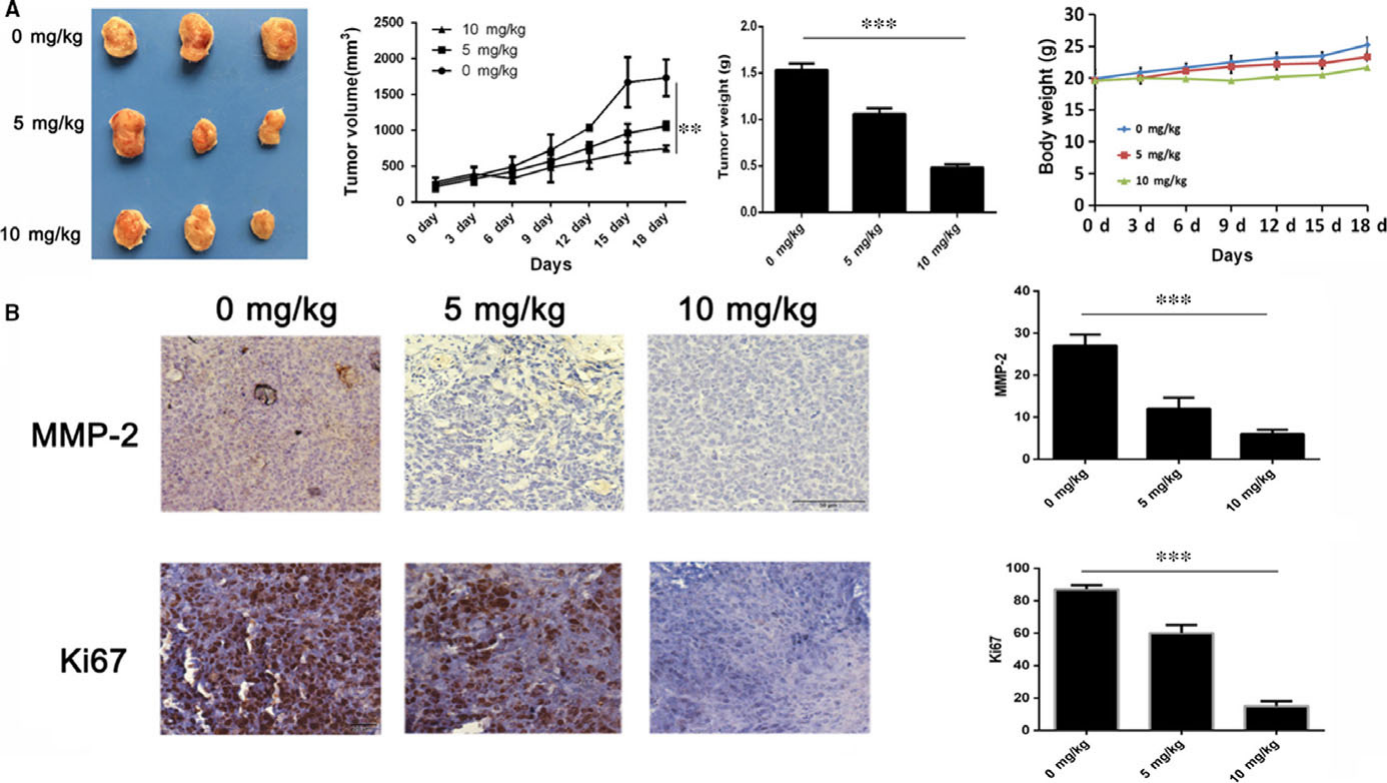
Anti‐tumour effects of BA in vivo. A, The tumour volumes and body weights were measured every 3 days, the tumour tissues were photographed and weighted at the end. B, The expression of Ki‐67 and MMP‐2 in the tumour sections determined by IHC staining (×100). *P <* *0.05, **P <* *0.01, ***P <* *0.001 vs Control (0 μm) group

The in vitro data suggested that BA showed a remarkable inhibitory effect on HCC cell lines proliferation, so we further investigated the anti‐tumour activity of BA in vivo. The HepG2 tumour‐bearing mice have received daily intraperitoneally injection (i.p.) of BA at the dose of 5 and 10 mg/kg for 18 d. The tumour volumes were measured and calculated every 3 days, at the end, the tumour tissues were photographed and weighted. As shown in Figure 5A, the tumours treated with both dosages of BA were obviously smaller than the control group visually, and the tumour volumes and weights results were consistent with the pictures. These results showed that BA has a strong anti‐tumour activity in vivo. In order to investigate the mechanisms underlying BA activity, Ki‐67 and MMP‐2, two markers of proliferation and apoptosis were determined by IHC. As shown in the Figure 5B, BA treatment significantly reduced the proliferating cells that stained positive for nuclear Ki‐67. The MMP‐2 positive cells were also decreased after treatment with BA. Overall, the results indicated that BA inhibits cell proliferation and induces apoptosis in the tumour tissues for inhibiting of Ki‐67 and MMP‐2.

### 3.6 BA inhibits HepG2 tumour pulmonary metastasis

**Figure 6:**
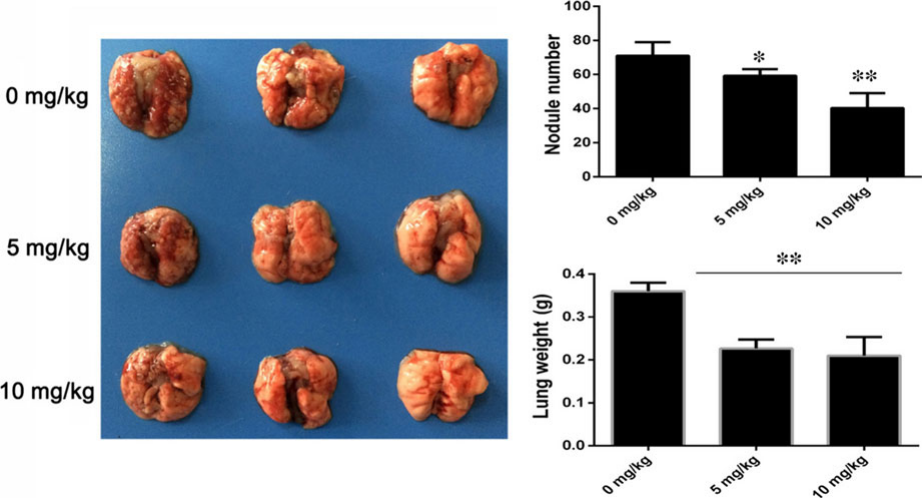
Betulinic acid inhibits pulmonary metastasis of HCC. The metastatic nodules were photographed and counted. BA reduced the metastatic nodules at 5 and 10 mg/kg significantly. The metastatic lungs were weighted. BA significantly decreased the lung weights at 5 and 10 mg/kg. *P <* *0.05, **P <* *0.01, ***P <* *0.001 vs Control (0 μm) group

Hepatocellular carcinoma (HCC) shows a high metastatic potential, and the lung is considered to be the most common target of metastasis.^30^ So the anti‐metastasis effect of BA was investigated in a pulmonary metastasis mice model. The results showed that multiple large metastatic nodules were observed in the vehicle group; however, the extents of metastatic nodules were markedly reduced in BA‐treated mice (*P *<* *0.05). Moreover, BA also decreased the lung weights significantly compared to the vehicle groups (*P *<* *0.01) (Figure 6). Overall, these results further demonstrated that BA shows an inhibitory effect on pulmonary metastasis of HCC.

### 3.7 Safety profile of BA

**Figure 7:**
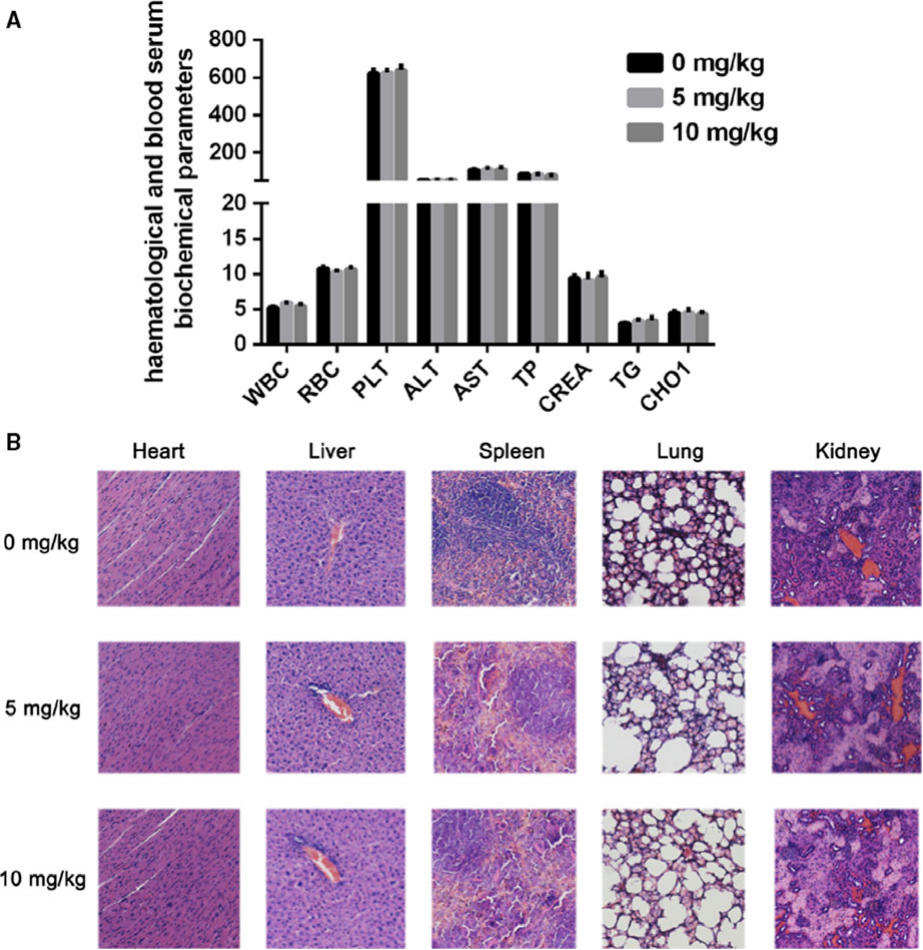
The safety profile evaluation of BA. A, Hematological and serum biochemistry analysis. WBC: white blood cell (10^9^/L); RBC: red blood cell (10^12^/L); PLT: blood platelet (10^9^/L); ATL: Glutamic pyruvic transaminase (U/L); AST: Aspartate transferase (U/L); TP: Total protein (g/L); CREA: Creatinie; TG: Triglyceride; CHOl: Cholesterol. B, The H&E staining of paraffin‐embedded sections of the heart, liver, spleen, and kidney (×100). *P <* *0.05, **P <* *0.01 vs Control (0 μm) group

In order to evaluate the safety profile of BA, some critical blood biochemical indicators and histopathologic changes of important organs were analysed after the tumour‐bearing experiment. As shown in Figure 7A, BA‐treated mice did not show any significant hematopathological changes. Similarly, the histopathologic analysis of heart, liver, spleen, lung, and kidney also did not show obvious pathologic changes in the BA‐treated mice compared to the control (0 μm) group (Figure 7B).

## 4 DISCUSSIONS

Hepatocellular carcinoma (HCC) is a high incidence and mortality malignant tumour globally.^31^ China, Southeast Asia, sub‐Saharan western, and eastern Africa showed the highest incidence of HCC according to GLOBOCAN 2012.^4^ In the early stage of HCC, resection and liver transplantation are recommended and will get survival and prognosis benefits, however, in the advanced HCC, the prognosis is poor because of the high reoccurrence rate and metastatic potential of HCC cells and still lack effective medicine to control the progression.^32^ Therefore, exploring for safe and effective novel agents with inhibition of tumour progression and metastasis are still a hot research topic. In the present study, we investigated the in vitro and in vivo effects of a natural product BA on HCC growth and tumour metastasis.

In recent years, BA has attracted increasing attention for its potent anti‐tumour properties. Our study suggested that BA inhibited the viability of three HCC cell lines including HepG2, LM3, and MHCC97H significantly. In addition, the proliferation inhibitory effect of BA against HepG2 and LM3 cells were further confirmed by clonogenic assay.

Apoptosis plays an important role in the elimination of damaged cells to maintain homeostasis in normal condition. However, defects in apoptotic signalling pathways could stimulate carcinogenesis and promote cancer cell survival.^33^ Therefore, inducing tumour cell apoptosis is one of the therapeutic approaches to treat cancers. Generally, two main major apoptotic pathways on the effector caspases are characterised: intrinsic (mitochondrial‐mediated) and extrinsic apoptosis pathways.^34^ The Bcl‐2 family proteins and caspase cascades play an important role in apoptosis regulation.^35^ Bax is a pro‐apoptotic protein in the mitochondrial outer membrane; it promotes intrinsic apoptosis for releasing of apoptogenic molecules, however, its pro‐apoptotic effect is blocked by Bcl‐2 by blocking Bax releasing and oligomerisation.^36^ Previous studies have suggested that BA shows remarkable pro‐apoptotic property for inducing mitochondrial‐mediated apoptosis.^37^ In this study, we evaluated the effect of BA on apoptosis and apoptosis‐related protein in the HCC cell lines. Our data indicated that that BA‐induced HepG2 cells apoptosis characterised condensed nuclei and nuclear fragmentation and increased the apoptosis rate. Moreover, BA increased the expression of pro‐apoptotic Bax and cleaved caspase‐3 and reduced the expression of anti‐apoptotic Bcl‐2.

Mitochondria transmembrane potential (Δψm) depolarisation would induce the apoptogenic factors to release from mitochondria into cytosol and loss of oxidative phosphorylation which ultimately induces apoptosis.^38^ Moreover, the permeability and polarisation of mitochondrial membrane were also regulated by Bcl‐2 family proteins.^39^ In the present study, BA treatment induced a loss of ΔΨm in HepG2 cells was observed. Furthermore, evidence has demonstrated that mitochondria are the source of ROS, excessive of ROS would disrupt ΔΨm and ultimately induce apoptosis.^40^ In this study, we demonstrated that BA treatment markedly reduces the levels of ROS in HepG2 cells.

The anti‐tumour activity of BA on HCC was confirmed in vitro, however, it does not imply that it has the same activity in vivo. So, we investigated the anti‐tumour activity of BA on an established HCC model in mice. The results demonstrated that BA treatment inhibited the tumour growth significantly characterised by reducing of the tumour volumes and weights. Meanwhile, BA treatment also decreased the expression level of Ki‐67 and MMP‐2 in tumour cells.

HCC is aggressive cancer with high invasion and metastasis potential which induce poor prognosis and dismal outcome. Cancer cell migration and invasion were demonstrated to be one of the pivotal steps in various cancers metastasis.^28^ So we investigated the inhibition effects of BA on migration and invasion of HepG2 cells. Our results demonstrated that BA significantly inhibited the migration and invasion of HepG2 cells. Moreover, BA also decreased the adhesive ratios (%) of HepG2 cell.

Extracellular matrix (ECM) and the basement membrane degradation induced by MMPs are essential for malignant tumour invasion and metastasis. The MMPs, especially MMP‐2 and MMP‐9, and TIMP2 have been implicated in invasion and metastasis of malignant tumour.^41^ Our data showed that BA treatment decreases the expression of MMP‐2 and MMP‐9, and increase the expression of TIMP2. Pulmonary metastasis is the most common type of metastasis in HCC, so we also investigated the anti‐metastasis effect of BA in a pulmonary metastasis animal model. As shown in the results, the anti‐metastasis effect of BA was confirmed in vivo. In all, these results demonstrated that BA shows an inhibitory effect on metastasis of HCC in vitro and in vivo. In addition, no obvious toxicity was observed during the BA treatment in mice.

In conclusion, we investigated the effect of BA on proliferation, apoptosis, invasion, and metastasis in HCC cell lines and on tumour growth and pulmonary metastasis in mice. The results suggested that BA inhibited the proliferation and induced apoptosis of HCC cells via the mitochondrial apoptotic pathway. Furthermore, BA also significantly inhibited HCC growth and blocked pulmonary metastasis in vivo by regulated the metastasis‐related proteins including MMP‐2, MMP‐9, and TIMP2 without obvious toxicity. Altogether, these data provided strong evidence that BA will has benefits for the recurrence and metastasis of HCC and may be a promising HCC drug candidate.

## CONFLICT OF INTEREST

The authors declare no conflicts of interest.
